# Chitosan Sensitivity of Fungi Isolated from Mango (*Mangifera indica* L.) with Anthracnose

**DOI:** 10.3390/molecules27041244

**Published:** 2022-02-12

**Authors:** Griselda Valenzuela-Ortiz, Soila Maribel Gaxiola-Camacho, Cesar San-Martín-Hernández, Miguel Ángel Martínez-Téllez, Emmanuel Aispuro-Hernández, Jaime Lizardi-Mendoza, Eber Addí Quintana-Obregón

**Affiliations:** 1 Facultad de Medicina Veterinaria y Zootecnia, Universidad Autónoma de Sinaloa, Culiacán 80260, Mexico; griseval1993@gmail.com (G.V.-O.); soilagaxiola@uas.edu.mx (S.M.G.-C.); 2 Colegio de Postgraduados, Campus Montecillo, Texcoco 56230, Mexico; sanmartin.cesar@colpos.mx; 3 Centro de Investigación en Alimentación y Desarrollo, A.C. Coordinación de Tecnología de Alimentos de Origen Vegetal, Hermosillo 83304, Mexico; eaispuro@ciad.mx; 4 Centro de Investigación en Alimentación y Desarrollo, A.C. Coordinación de Tecnología de Alimentos de Origen Animal, Hermosillo 83304, Mexico; jalim@ciad.mx; 5 Programa de Investigadoras e Investigadores por México del CONACYT-Centro de Investigación en Alimentación y Desarrollo, A.C. (CIAD), Coordinación de Tecnología de Alimentos de Origen Vegetal, Hermosillo 83304, Mexico

**Keywords:** phytopathogen, *Colletotrichum* complex, antifungal, chitosan

## Abstract

In Mexico, the mango crop is affected by anthracnose caused by *Colletotrichum* species. In the search for environmentally friendly fungicides, chitosan has shown antifungal activity. Therefore, fungal isolates were obtained from plant tissue with anthracnose symptoms from the state of Guerrero in Mexico and identified with the ITS and β-Tub_2_ genetic markers. Isolates of the *Colletotrichum gloeosporioides* complex were again identified with the markers ITS, Act, β-Tub_2_, GADPH, CHS-1, CaM, and ApMat. Commercial chitosan (Aldrich, lot # STBF3282V) was characterized, and its antifungal activity was evaluated on the radial growth of the fungal isolates. The isolated anthracnose-causing species were *C. chrysophilum*, *C. fructicola*, *C. siamense*, and *C. musae*. Other fungi found were *Alternaria* sp., *Alternaria tenuissima*, *Fusarium* sp., *Pestalotiopsis* sp., *Curvularia lunata*, *Diaporthe pseudomangiferae*, and *Epicoccum nigrum*. Chitosan showed 78% deacetylation degree and a molecular weight of 32 kDa. Most of the *Colletotrichum* species and the other identified fungi were susceptible to 1 g L^−1^ chitosan. However, two *C. fructicola* isolates were less susceptible to chitosan. Although chitosan has antifungal activity, the interactions between species of the *Colletotrichum gloeosporioides* complex and their effect on chitosan susceptibility should be studied based on genomic changes with molecular evidence.

## 1. Introduction

Diseases caused by phytopathogenic fungi during pre- and post-harvest storage leads to significant losses for farmers and generate conditions for food insecurity [1]. Farmers have managed to minimize losses in the production of horticultural products with the use of agrochemicals, for example, fungicides such as azoxystrobin, fludioxonil, captan, merivon, imazalil, propiconazole, fosetyl-Al, orthophenylphenol, prochloraz, pyrimethanil, methylthiophanate, thiabendazole, and fludioxonil, among others [1,2,3,4]. However, some of the disadvantages of using these products are the resilience that fungi may develop [5], the damage to health, and damage to the environment [6]. This highlights the need to control post and pre-harvest diseases caused by phytopathogens with compounds that contribute to the success of sustainable agriculture and reduce the use of harmful agrochemicals [7]. The development of alternatives to traditional fungicides aims to reduce environmentally harmful products to control phytopathogenic fungi [8]. In this regard, some compounds of natural origin, such as essential oils, methanolic extracts, plant extracts, lipoproteins, and chitosan, have shown antifungal effects [7,9,10].

Chitosan is the direct derivative of chitin; it is a natural, biodegradable, non-toxic compound with fungicidal effects that induce defense mechanisms in plant tissues [11]. Likewise, chitosan has been evaluated in phytopathogenic fungi showing its antifungal activity against *Fusarium*, *Rhizopus*, *Aspergillus*, *Alternaria*, and *Colletotrichum* [12,13,14,15]. The benefits of chitosan in agriculture encourage its use for the pre- and post-harvest control of horticultural fruits [16]. However, the sensitivity of the different fungal strains to chitosan often varies according to intrinsic characteristics proper of each species, e.g., particularities in the cell wall and membrane composition.

One of the most important fruits in Mexico is the mango (*Mangifera indica* L.), the 2019-year production of 2,089,041 t positioned Mexico as the sixth producer of mango worldwide, where the state of Guerrero is one of the leading producers nationwide [17]. However, farmers in Mexico still report losses related to various fungal diseases, one of them being anthracnose caused by fungi of the *Colletotrichum* genus [18,19]. Earlier studies have shown the antifungal effect of chitosan on *Colletotrichum* isolates [20], most of them identified as part of the *Colletotrichum* complexes; nevertheless, there is still little information on the chitosan sensitivity at the level of the *Colletotrichum* species. A *Colletotrichum* complex requires identification with the genomic alignment of at least one gene, while species identification requires at least three genes [21]. This research aimed to evaluate the in vitro chitosan sensitivity of fungal isolates obtained from anthracnose injuries in mango from Guerrero, Mexico. The species were identified with seven genes using a genomic alignment approach.

## 2. Results and Discussion

### 2.1. Identification of Fungal Isolates

**Figure 1:**
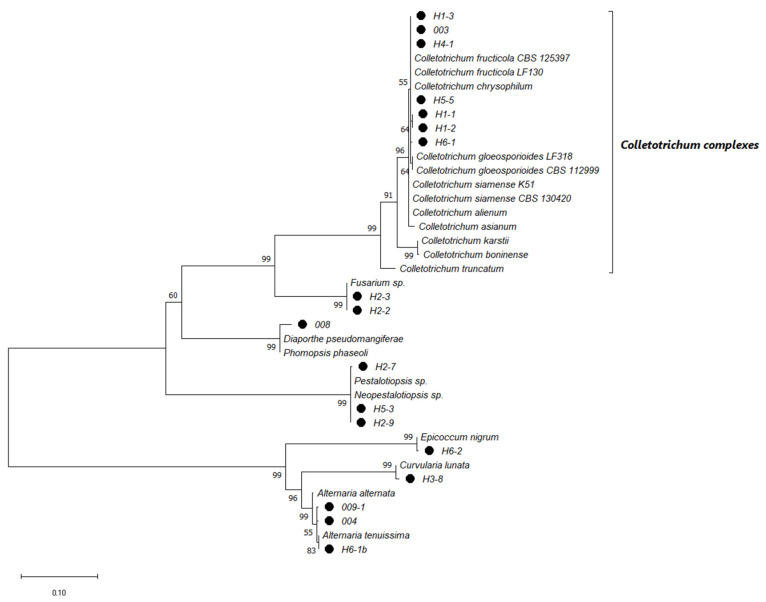
Tree phylogenetic relationship amongst fungi isolated from *Mangifera indica* L. with anthracnose-like symptoms. Maximum likelihood tree based on combined ITS and β-Tub_2_ sequence data. Black dots show isolates characterized in the present work. Bootstrap support values are displayed at the tree nodes, and the branch lengths and scale bar represent the number of substitutions per site. ^●^ Isolates from Guerrero, Mexico.

**Figure 2:**
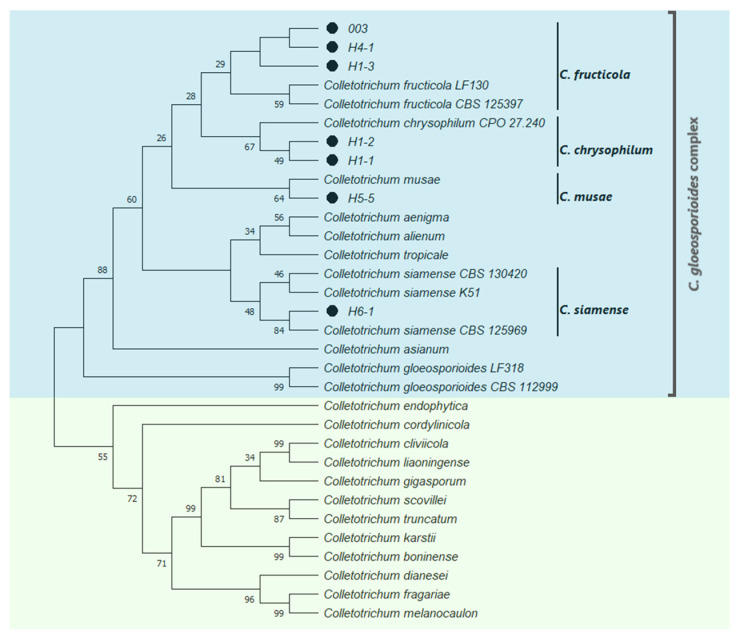
Identification of *Colletotrichum* species isolated from *Mangifera indica* L. Bootstrap consensus tree inferred by the maximum likelihood method and general time-reversible model based on the sequences of ITS, Act, β-Tub_2_, GAPDH, CHS-1, CaM, and ApMat. Bootstrap values next to the branches stand for the percentage of replicate trees in which the associated taxa clustered together (1000 replicates). Black dots show isolates characterized in the present work.

The sequences of ITS and β-Tub_2_ (first genomic alignment) allowed us to classify the fungal isolates obtained from leaves and fruit into seven main clades consistent of different fungal genera, in which seven isolates belong to the *C. gloeosporioides* complex, the causal agent of anthracnose in mango (Figure 1). Likewise, the non-*Colletotrichum* fungal isolates found in the present work belong to species associated with mango infections (Figure 1). The second genomic alignment using ITS, Act, β-Tub_2_, GAPDH, CHS-1, CaM, and ApMat sequences from the isolates of the *Colletotrichum* complex, allowed the identification of four species of the *Colletotrichum* genera (Figure 2).

Among the isolates belonging to the *Colletotrichum* complex species, only one was isolated from fruit, while the rest were found in infected leaves. The complexes of *C. gloeosporioides* are more adapted to infect vegetative tissues than fruit, contrasting, for example, with the *C. acutatum* complex, which is more adapted to fruit infection [21]. Concerning the fungal identification of mango isolates, earlier Tovar-Pedraza et al. [22], obtained isolates from the *C. gloeosporioides* complex, and found the species *C. alienum*, *C. asianum*, *C. siamense*, and *C. tropicale* using Apn2/MAT intergenic spacer sequences. In contrast, our results suggest the species *C. fructicola*, *C. chrysophilum*, *C. musae*, and *C. siamense* as causal agents of anthracnosis in mango. These differences may be related to the sample size and the goals of each study. Tovar-Pedraza et al. [22] obtained samples from eight Mexican states (Sinaloa, Nayarit, Colima, Michoacan, Guerrero, Oaxaca, Chiapas, and Veracruz); their goal was to find the distribution of *Colletotrichum* species in mango, while our study considered samples from one Mexican state (Guerrero) to obtain *Colletotrichum* isolates from mango for evaluating the sensitivity to chitosan. Additionally, Li et al. [23] reported *C. asianum*, *C. fructicola*, and *C. siamense* on mango in China. Studies on anthracnose disease in mango have shown that the *Colletotrichum* species belong to the *C. gloeosporioides* complex, and multiple markers are necessary for proper species identification [24].

In addition to the *Colletotrichum* species related to anthracnose in mango, the other identified fungal isolates belong to six different genera, including *Alternaria*, *Fusarium*, *Pestalotiopsis*, *Curvularia*, *Diaporthe*, and *Epicoccum* (Figure 1). These fungi can act as saprophytes and have been reported to occasionally cause diseases in mango, with some symptoms such as anthracnose [25,26,27,28,29,30].

### 2.2. Chitosan Characterization and Sensitivity of Isolated Fungi

**Figure 3:**
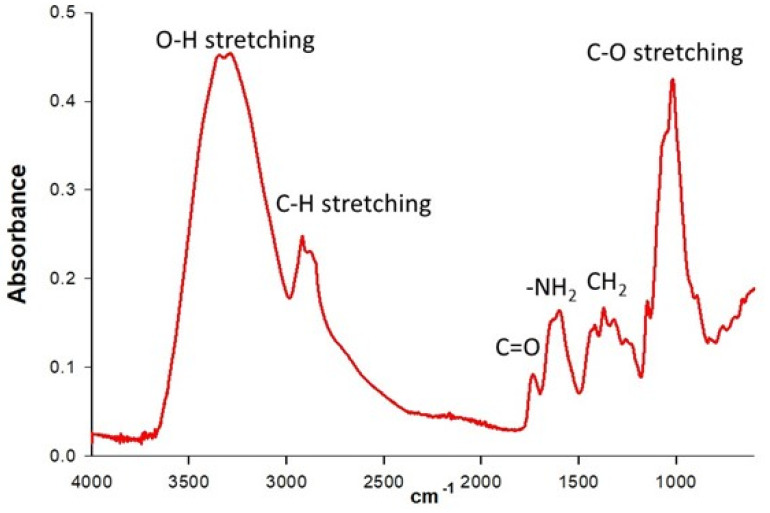
FT-IR spectrum corresponding to the chitosan sample.

The FT-IR spectrum corresponding to the chitosan sample (Figure 3) allowed calculating 78.5 ± 0.1% degree of deacetylation, and the molecular weight was 32.0 ± 6.4 kDa using a capillary viscosimeter. Usually, chitosans at the 10–200 kDa range are considered low molecular weight [31]. The degree of deacetylation and the molecular weight is related to the type of biological activity of the chitosan with the fungus and is fully documented; Grande-Tovar et al. [32] summarize the three main mechanisms proposed in the last few years: 1) interaction between amino groups of chitosan with anionic groups on the cell wall surface; 2) interaction of the positive amino groups of chitosan with the negative charges of phospholipids; and 3) binding of DNA.

**Figure 4:**
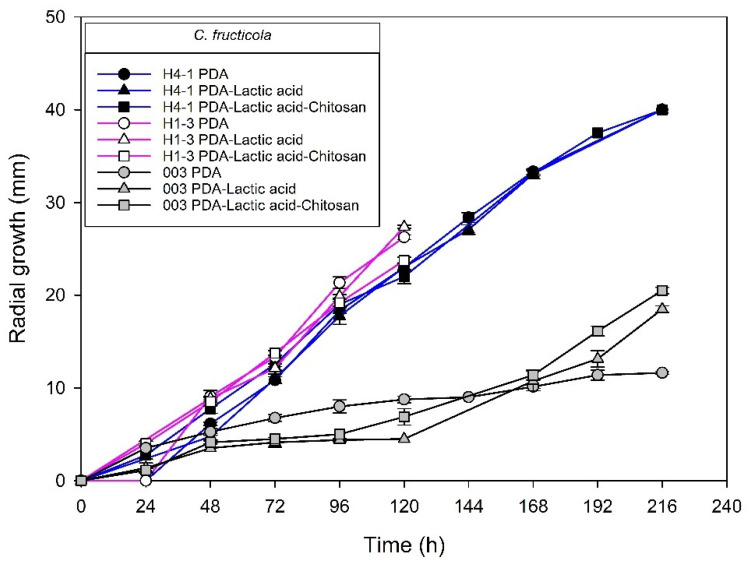
*Colletotrichum fructicola* species isolated from anthracnose of mango (H4-1, H1-3, and 003). Kinetics of radial growth (mm) in medium PDA, PDA-lactic acid (0.05 M), and PDA-lactic acid (0.05 M) with 1 g L^−1^ chitosan at 25 °C.

**Figure 5:**
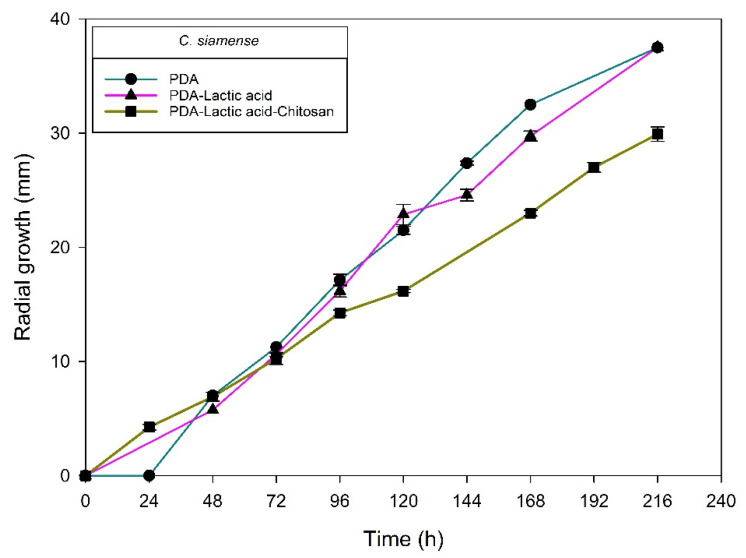
*Colletotrichum siamense* isolated (H6-1) from anthracnose of mango. Kinetics of radial growth (mm) in medium PDA, PDA-lactic acid (0.05 M), and PDA-lactic acid (0.05 M) with 1 g L^−1^ chitosan at 25 °C.

**Figure 6:**
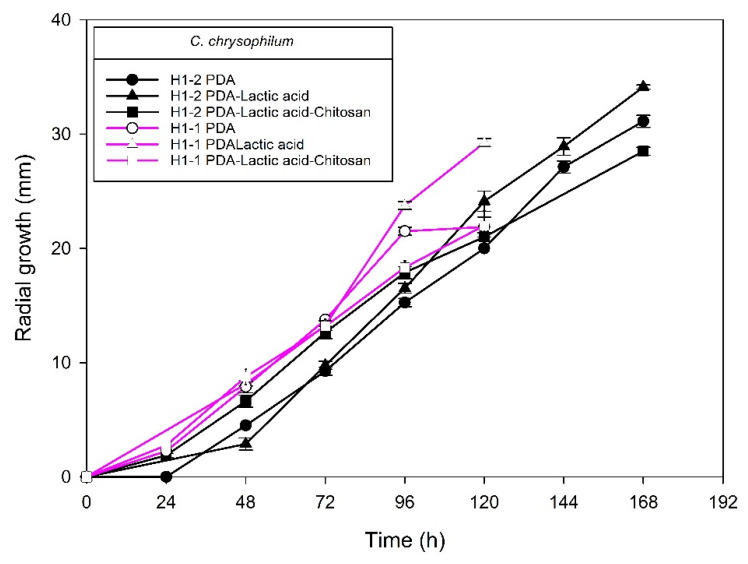
*Colletotrichum chrysophilum* isolates from anthracnose of mango (H1-2 and H1-1). Kinetics of radial growth (mm) in medium PDA, PDA-lactic acid (0.05 M), and PDA-lactic acid (0.05 M) with 1 g L^−1^ chitosan at 25 °C.

**Figure 7:**
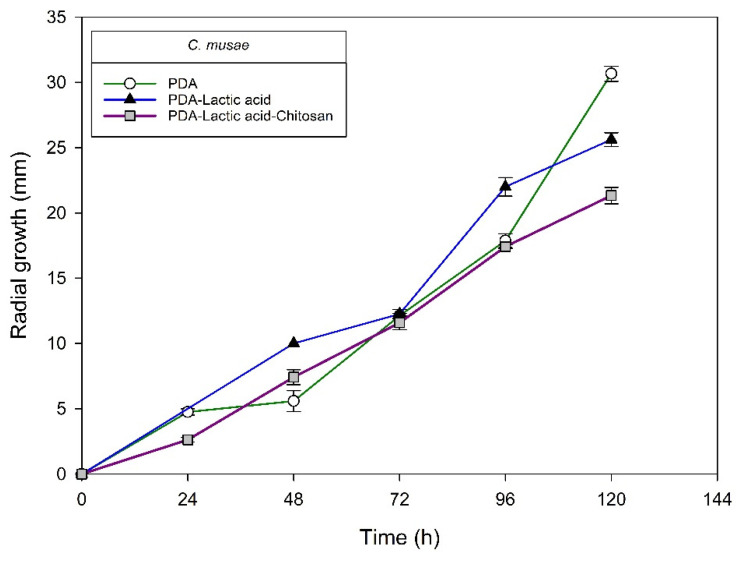
*Colletotrichum musae* isolated from anthracnose of mango. Kinetics of radial growth (mm) in medium PDA, PDA-lactic acid (0.05 M), and PDA-lactic acid (0.05 M) with 1 g L^−1^ chitosan at 25 °C.

Figure 4, Figure 5, Figure 6 and Figure 7 show the radial growth kinetics of *Colletotrichum* isolates in medium PDA, PDA-lactic acid (0.05 M), and PDA-lactic acid (0.05 M) with 1 g L^−1^ of chitosan at 25 °C.

The isolate H1–3 was sensitive, but *C. fructicola* isolates H4-1 and 003 were less sensitive to 1 g L^−1^ chitosan (Figure 4, Table 1). All the other *Colletotrichum* isolates were sensitive to chitosan (Table 1). 

The *C. fructicola* isolate 003 was the only one obtained from the fruit. Its high growth on PDA-lactic acid contrasts with the growth on PDA because it may be adapted to develop in tissues with organic acids present (such as those present in the fruit). Lactic acid can sham the organic acids present in the fruit. The absence of these in the artificial PDA medium without the addition of lactic acid can be a factor that affects mycelial development. However, there are not enough data to hypothesize what happens with this isolate since it is the only one obtained from the fruit. 

Table 1 shows the radial growth rates in the log phase and the percentage of radial inhibition at 120 h of the *Colletotrichum* species causing anthracnose on mango from Mexico to chitosan at a concentration of 1 g L^−1^. Most isolates were susceptible to chitosan except for two *C. fructicola* specimens.

The effect of chitosan on fungal biological systems related to molecular weight (low, medium, or high) is well known. Low-molecular-weight chitosan can be more effective against mycelial growth [31]. Additionally, other studies have suggested that concentration may also be a factor that generates diverse defense responses in fungi. In general, over 1 g L^−1^ of chitosan inhibits 80–100% of the fungal growth [33,34], and it has complete in vitro inhibition from 10 g L^−1^ [35]. However, after several hours, growth recovers [36]. In contrast, low concentrations of chitosan (1 g L^−1^ and below) inhibit fungal growth [37,38,39], but there are other effects on the cell at low concentrations. Chitosan binds to the negatively charged cell surface, disturbs the cell membrane, inducing leakage of intracellular components [40], stimulates respiration, and produces the efflux of significant amounts of cations [36]. In *Colletotrichum* species, inhibitions of 25% to concentrations of 1 g L^−1^ have a fungistatic effect [41]. In this study, the antifungal capacity on mycelial growth was evaluated at concentrations of 0.1 to 1 g L^−1^ to cause a moderate attack of chitosan on fungal cells or fungistatic activity (not total inhibition or fungicide activity). Chitosan inhibited most of the isolates exposed to 0.75 and 1 g L^−1^ of chitosan (Table 2). The results of the other concentrations tested have been included in Table 2. The range of chitosan concentrations and inhibitions were insufficient to be able to estimate the MIC against isolated *Colletotrichum*. Adjusting final concentrations of chitosan at 10.0, 5.0, 2.5, 1.25, 0.625, 0.312 and 0.0 g L^−1^ can estimate the MIC for *Colletotrichum gloeosporioides* [42].

In the *Colletotrich*um isolates, the causal agent of anthracnose, it was possible to differentiate less susceptible strains of the same species at low concentrations (H4-1 and 003). This fact is relevant for future studies in our research group to elucidate the mechanisms of susceptibility or the possible resistance of *Colletotrichum* isolates to chitosan and should be studied based on genomic changes with molecular evidence.

The percentage of radial growth inhibition at a 1 g L^−1^ concentration of chitosan was greater than 10%, and the radial growth rates of the log phase with respect to PDA-acid were reduced except for H4-1 and 003 isolates (Table 1). Earlier studies have shown the effect of chitosan on the radial growth of *Colletotrichum* species. Ramos et al. [43] reported radial growth inhibition at concentrations higher than 5 g L^−1^ chitosan with 40 kDa molecular weight and 85% DD in *C. asianum*, *C. fructicola*, *C. tropicale*, and *C. siamense* species.

Radial extension rate decreased in most of the *Colletotrichum* species growing in vitro with chitosan. The growth of fungi includes four distinctive phases: the lag phase (I), the log phase (II), the slow down phase (III), and the steady growth phase (IV) [44]. During the log phase of balanced growth, the mycelium of fungi undergoes primary metabolism [45], so a decrease in rate is indicative of fungistatic activity by inhibition of the primary metabolism. However, radial growth of the *C. fructicola* isolate H4-1 from leaves was less inhibited, while 003 from the fruit was not inhibited with 1 g L^−1^ chitosan at 120 h; their log phase radial extension rates were not affected by the low molecular weight chitosan. No earlier studies were found in which the effect of chitosan was evaluated on C*. fructicola* isolates identified to the species level using more than three genetic markers, which is necessary to ensure proper identification to the species level in the genus *Colletotrichum*. Ramos et al. [43] reported inhibition of *C. fructicola* at a 5 g L^−1^ chitosan concentration; however, this species was one of the less inhibited by chitosan, although it showed hyphae with granular and corrugated surface when exposed to chitosan. The lower inhibition that chitosan exerted on isolates H4-1 and 003 may also be related to the virulence of the fungus; for instance, *C. fructicola* was more aggressive than *C. siamense* on peach [46], but in mango from Mexico, it has been reported that *C. siamense* and *C. asianum* have higher virulence than *C. fructicola* [22]. These variations in the degree of virulence of the fungus with its host can also be reflected in the sensitivity to fungicides that are applied, so one of the aspects to consider for the control of *Colletotrichum* complex species is the execution of fungicide sensitivity test [21] with a species-level identification using more than three genetic markers. In our study, two strains of *C. fructicola* were less susceptible. Therefore, it would be interesting to evaluate if there is a relationship between the degree of virulence (high or low) in mango and the sensitivity to chitosan as a hypothesis to be tested in future studies.

**Figure 8:**
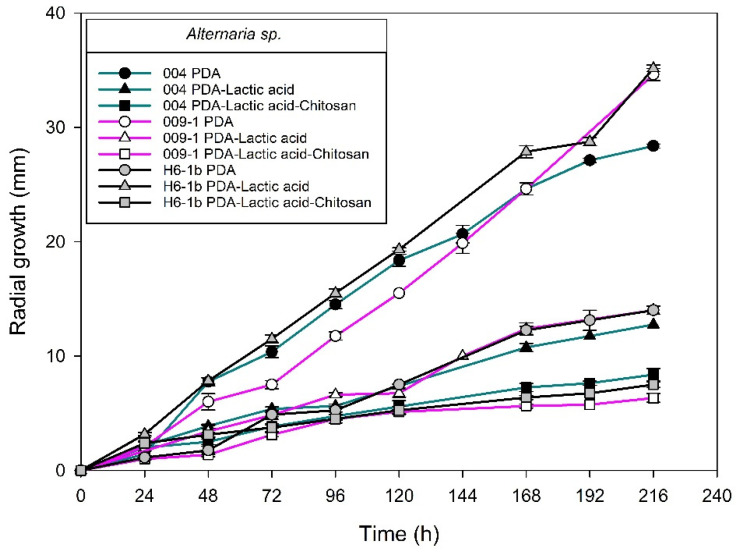
*Alternaria* sp. isolated from anthracnose of mango. Kinetics of radial growth (mm) in medium PDA, PDA-lactic acid (0.05 M), and PDA-lactic acid (0.05 M) with 1 g L^−1^ chitosan at 25 °C.

**Figure 9:**
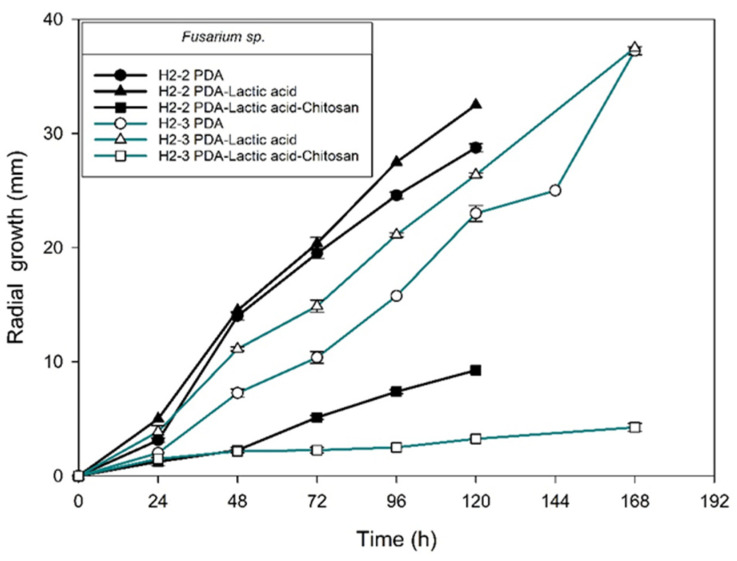
*Fusarium* sp. isolates from anthracnose of mango. Kinetics of radial growth (mm) in medium PDA, PDA-lactic acid (0.05 M), and PDA-lactic acid (0.05 M) with 1 g L^−1^ chitosan at 25 °C.

**Figure 10:**
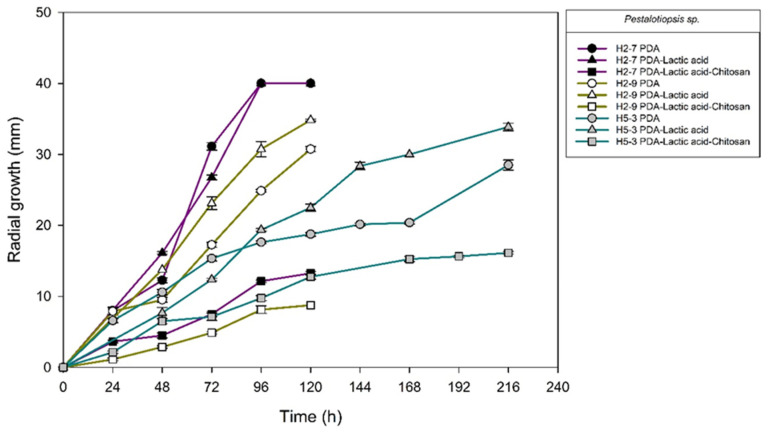
*Pestalotiopsis* sp. isolates from anthracnose of mango. Kinetics of radial growth (mm) in medium PDA, PDA-lactic acid (0.05 M), and PDA-lactic acid (0.05 M) with 1 g L^−1^ chitosan at 25 °C.

**Figure 11:**
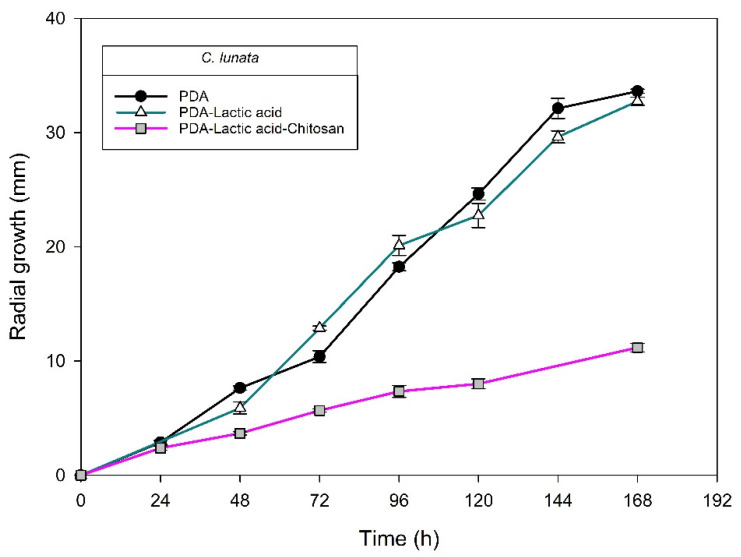
*Curvularia lunata* isolated from anthracnose of mango. Kinetics of radial growth (mm) in medium PDA, PDA-lactic acid (0.05 M), and PDA-lactic acid (0.05 M) with 1 g L^−1^ chitosan at 25 °C.

**Figure 12:**
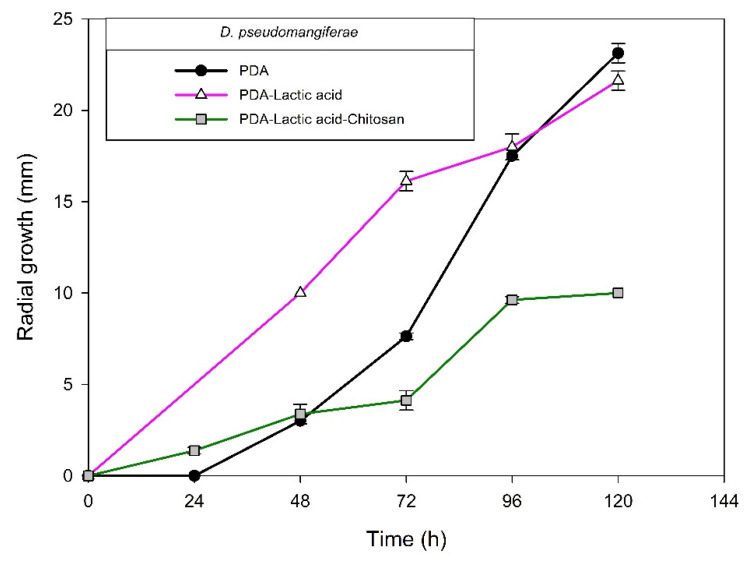
*Diaporthe pseudomangiferae* isolated from anthracnose of mango. Kinetics of radial growth (mm) in medium PDA, PDA-lactic acid (0.05 M), and PDA-lactic acid (0.05 M) with 1 g L^−1^ chitosan at 25 °C.

**Figure 13:**
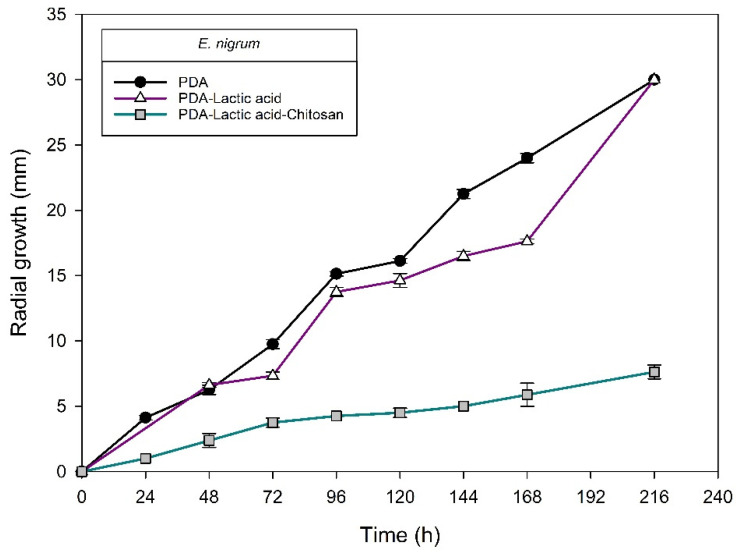
*Epicoccum nigrum* isolated from anthracnose of mango. Kinetics of radial growth (mm) in medium PDA, PDA-lactic acid (0.05 M), and PDA-lactic acid (0.05 M) with 1 g L^−1^ chitosan at 25 °C.

Concerning the fungi that did not belong to the *Colletotrichum* genus, all the isolates found were susceptible to chitosan (Table 3; Figure 8, Figure 9, Figure 10, Figure 11, Figure 12 and Figure 13 and Table 2). In Mexico, anthracnose is the primary disease caused by *Colletotrichum* that affects mango crops, but it is not discarded that the infections caused by other fungal genera can generate problems for mango production; in that case, chitosan may be an alternative to evaluate. Furthermore, the presence of these fungi could affect the fruit quality by worsening necrotic signs at sites initially injured by *Colletotrichum*.

Fungal isolates of plant tissue with anthracnose in mango from Mexico belong to the *Colletotrichum* complex. The use of seven genetic markers in the genomic alignment identified the species *C. fructicola, C. musae*, and *C. chrysophilum*. The fungi of the *Colletotrichum* complex are susceptible to chitosan except for two isolates of the species *C. fructicola* that showed less susceptibility to chitosan. Likewise, the genera *Alternaria*, *Fusarium*, *Pestalotiopsis*, *Curvularia*, *Diaporthe*, and *Epicoccum*, which cause other diseases in mango, showed susceptibility to chitosan in all cases. Therefore, chitosan is an alternative to be evaluated in the control of anthracnose and other fungal infections in mango. However, due to the demonstrated lower susceptibility to chitosan presented by a *C. fructicola* specimen, the interactions between the species of the complex of *C. gloeosporioides* in anthracnose and their effect on the susceptibility or resistance to chitosan must be considered.

## 3. Materials and Methods

### 3.1. Identification of Fungal Isolates

Colletotrichum isolates were obtained in the 2019 agricultural cycle from Cuajinicuilapa, Guerrero, Mexico. Mango leaves with anthracnose symptoms from the lower foliage of the tree were cut from the petiole and stored individually on paper towels. A leaf with anthracnose disease contains black necrotic spots of irregular shape on both sides of the leaf. The infected leaves were transferred to the laboratory at room temperature to obtain fungal isolates. Likewise, samplings were carried out on commercial maturity mango fruit with anthracnose from Guerrero. A mango with anthracnose shows deep, prominent, and generally rounded dark brown to black spots [20,47]. From the infected leaves and fruits, 0.5 × 0.5 cm tissue sections cuts were obtained and disinfected with sodium hypochlorite (NaOCl) 0.5% (*v*/*v*) for 2 min; then they were washed with sterile distilled water and dried with sterile filter paper. Each fragment was individually deposited in the center of a Petri dish with culture medium Potato Dextrose Agar (PDA) and incubated at 25 °C in the absence of light until the progress of mycelium for 8–10 days.

DNA extraction was carried out from mycelium of each colony, a sterile scalpel was used to obtain 100 mg of mycelium, and it was placed in Eppendorf tubes (5 Ml). The mycelium breaking was carried out through three methods; in the first method, the mycelium sample was crushed by liquid nitrogen using a mortar; in the second, glass beads and vortex were used, crushing the mycelium for 1 min, and the third method was using a grinder with pellet pestles. The third method resulted in better DNA extraction yields. The fungal isolates were identified by a multi-locus sequence analysis scheme based on five genes (actin, Act; beta-tubulin 2, β-Tub_2_; glyceraldehyde-3-phosphate dehydrogenase, GAPDH; chitin synthase 1, CHS-1; calmodulin, CaM) and the ribosomal internal transcribed spacer (ITS) and Apn2-Mat1-2 (ApMat) intergenic spacer regions. Genomic DNA was extracted with the Plant/Fungi DNA Isolation Kit (Norgen Biotek Corporation, Canada) following the manufacturer’s instructions and used as a template for PCR reactions using the GoTaq^®^ Flexi DNA Polymerase (Promega, USA) and specific primers for each gene (Table 4).

The PCR products were purified with GFX columns (Amersham Bio-sciences, Piscataway, NJ, USA) and sequenced at Macrogen Inc. (Seoul, Korea). Sequences were analyzed against the GenBank database with the Blast tool (https://blast.ncbi.nlm.nih.gov/Blast.cgi), and the DNA sequences of the top hit matches were used as reference organisms for phylogenetic analysis. DNA sequence alignments were performed with the Clustal W function. Two concatenated phylogenetic trees were constructed with MEGA-X software v. 10.2.6. using the maximum-likelihood method and the general time-reversible model with gamma distribution and proportion of invariable sites (GTR + G + I) to estimate the evolutionary distances (1000 bootstrap replicates) [49,51]. For the first one, the combined sequences of ITS and β-Tub_2_ were used to determine the genus of the fungal isolates. In contrast, the second tree was constructed using ITS, Act, β-Tub_2_, GAPDH, CHS-1, CaM, and ApMat sequences that were directed to characterize the members of the *Colletotrichum* complexes, which are associated with anthracnosis disease in *Mangifera indica* L.

### 3.2. Chitosan Characterization and Sensitivity of Isolated Fungi

Low-molecular-weight chitosan (Aldrich, lot # STBF3282V, Saint Louis, MO, USA) was mixed and triturated with 120 mg of KBr for 10 min. The mixture was compacted with a hydraulic press (8 tons of pressure for 16 h), and the formed tablet was analyzed using a Fourier transformed infrared (FT-IR) resonance spectrometer (Spectrum GX FT-IR System, Perkin Elmer ™, Shelton, CT, USA). The spectrum obtained was within the frequency range of 4000–400 cm^−1^ [52]. The DD was calculated by the method proposed by Brugnerotto et al. [53] using reference baselines in the FT-IR spectrum to 1320 and 1420 cm^−1^ with equations 1 and 2 of Table 4.

The molecular weight (kDa) was obtained using Mark–Houwink–Sakurada (Table 5) where [ƞ] is the intrinsic viscosity of the polymer, Mv average molecular weight in Da (g mol^−1^), k = 0.070 g ml^−1^, and α = 0.81 according to the DD of chitosan [54,55]. The intrinsic viscosity was calculated by extrapolating to zero concentration of the Huggins, and Kraemer equations (Table 4) [56,57], using an Ubbelohde viscosimeter (0B-L123, CANNON) submerged in a water bath recirculation system at a constant temperature of 25 °C. Chitosan solutions were prepared with concentrations of 0.003–0.002 g L^−1^ [54], using a solution of 0.3 M acetic acid and 0.2 M sodium acetate as a solvent [58].

The chitosan activity in mycelial growth was evaluated over the fungal isolates obtained. A chitosan solution in lactic acid (0.05 M) was prepared; the pH was adjusted with NaOH (1 N) to 5.6 and sterilized for 15 min at 121 °C. Likewise, a culture medium with potato dextrose agar (PDA) was prepared and sterilized. At 45 °C, the chitosan solution and the PDA culture medium were mixed, and 20 mL were poured into sterile Petri dishes and cooled until solidified. The treatments obtained were PDA culture medium, acidified PDA culture medium (0.05 M lactic acid), and acidified PDA culture medium (0.05 M lactic acid) with chitosan (0.1–1 g L^−1^). Spores from colonies of 10 days of growth were extracted with a sterile microbiological loop and inoculated to the center of the medium culture within the Petri dish. It was incubated at 25 °C and colony diameters were manually measured every 24 h until the colony covered 80–90% of the surface of the plate. Subsequently, logarithmic growth kinetics were obtained (logarithm of the radius of the colony vs. time), and the log phase (exponential growth) was later used to calculate the radial growth speed of this growth stage [44]. The log phase is the most suitable stage for testing the susceptibility of filamentous fungi to antifungal compounds [59]. Likewise, the percentage of radial growth inhibition of the acidified PDA treatment with chitosan (0.1–1 g L^−1^) was calculated with respect to the acidified PDA culture medium. Finally, the strains that showed sensitivity and resistance to chitosan were scored.

The experimental study design was completely randomized; the study factor was isolated fungi and culture media composition. Analyses of variance (ANOVA) and Tukey tests, with the significance level set at *p* < 0.05, were carried out using JMP version 5.0 (SAS Institute Inc., Cary, NC, USA).

## Figures and Tables

**Table 1:** Chitosan against *Colletotrichum gloeosporioides* complex species isolated from anthracnose of mango. Rate of growth during log phase and growth radial inhibition in medium PDA, PDA-lactic acid (0.05 M), and PDA-lactic acid (0.05 M) with 1 g L^−1^ chitosan at 25 °C.

Isolate/Species	Rate of Growth in Log Phase μm h^−1^			Inhibition of Radial Growth ^1^ (%) by Chitosan at 120 h
	PDA	PDA-Lactic Acid	PDA-Lactic Acid-Chitosan	
H4-1/*C. fructicola*	212 ± 3 ^CDa2^	208 ± 3 ^Da^	202 ± 4 ^ABa^	2.84 ± 2.30 ^D^
H1-3/*C. fructicola*	248 ± 2 ^Bb^	268 ± 3 ^Ba^	217 ± 2 ^Ac^	13.38 ± 1.28 ^C^
003/*C. fructicola*	30 ± 4 ^Fb*2*^	117 ± 5 ^Fa^	126 ± 0 ^Da^	0.00 ± 0.00 ^D^
H6-1/*C. siamense*	175 ± 4 ^Ea^	173 ± 3 ^Ea^	135 ± 4 ^CDb^	29.95 ± 0.62 ^A^
H1-2*/C. chrysophilum*	228 ± 0 ^Cb^	242 ± 0 ^Ca^	149 ± 2 ^Cc^	12.95 ± 1.46 ^C^
H1-1*/C. chrysophilum*	207 ± 6 ^Db^	307 ± 1 ^Aa^	187 ± 7 ^Bc^	22.43 ± 1.20 ^B^
H5-5/*C. musae*	341 ± 5 ^Aa^	236 ± 5 ^Cb^	198 ± 4 ^Bc^	18.44 ± 1.37 ^B^

PDA: potato dextrose agar. ^1^ With respect to PDA-lactic acid (0.05 M). ^2^ Means ± standard deviation (*n* = 3). Values followed by different capital letters in columns and lowercase letters in rows are statistically different (Tukey *p* ≤ 0.05).

**Table 2:** Radial growth inhibition of *Colletotrichum gloeosporioides* complex isolates and other genera isolates from mango (PDA-lactic acid-chitosan) at 25 °C.

Isolate/Species	Radial Growth Inhibition^1^ (%) by Chitosan (g L^−1^) at 120 h			
	0.1	0.5	0.75	1
H4-1/*C. fructicola*	13.85 ± 0.93 ^2^	12.27 ± 1.08	0.00 ± 0.00	2.84 ± 2.30
H1-3/*C. fructicola*	0.00 ± 0.00	10.09 ± 0.70	12.84 ± 1.29	13.38 ± 1.28
003/*C. fructicola*	0.00 ± 0.00	0.00 ± 0.00	0.00 ± 0.00	0.00 ± 0.00
H6-1/*C. siamense*	6.00 ± 0.54	12.56 ± 0.28	23.44 ± 2.95	29.95 ± 0.62
H1-2/*C. chrysophilum*	40.89 ± 2.16	26.40 ± 1.23	42.99 ± 0.62	12.95 ± 1.46
H1-1/*C. chrysophilum*	0.00 ± 0.00	20.08 ± 0.36	19.22 ± 0.371	22.43 ± 1.20
H5-5/*C.musae*	0.00 ± 0.00	13.16 ± 0.41	11.69 ± 1.13	18.44 ± 1.37
004/*Alternaria* sp.	0.00 ± 0.00	18.67 ± 2.84	19.31 ± 0.97	20.28 ± 2.40
009-1/*Alternaria* sp.	0.00 ± 0.00	37.08 ± 1.94	33.37 ± 1.74	24.07 ± 2.62
H6-1b/*Alternaria tenuissima*	0.00 ± 0.00	68.83 ± 0.00	68.18 ± 2.75	72.72 ± 1.84
H2-2/*Fusarium* sp.	0.00 ± 0.00	53.46 ± 0.54	39.61 ± 0.54	71.54 ± 0.00
H2-3/*Fusarium* sp.	38.39 ± 0.92	77.25 ± 0.15	81.51 ± 0.54	87.62 ± 0.00
H2-7/*Pestalotiopsis* sp.	0.00 ± 0.00	54.68 ± 0.44	67.81 ± 1.32	67.18 ± 0.44
H2-9/*Pestalotiopsis* sp.	0.00 ± 0.00	62.23 ± 2.54	59.71 ± 2.03	74.87 ± 0.00
H5-3/*Pestalotiopsis* sp.	16.28 ± 0.53	49.63 ± 0.62	54.49 ± 0.07	43.33 ± 1.57
H3-8/*Curvularia lunata*	0.00 ± 0.00	50.53 ± 0.75	59.33 ± 0.34	65.22 ± 1.88
008/*Diaporthe pseudomangiferae*	0.00 ± 0.00	45.66 ± 0.30	60.72 ± 2.30	53.75 ± 0.0
H6-2/*Epicoccum nigrum*	10.26 ± 0.37	44.42 ± 0.80	59.82 ± 0.24	69.22 ± 1.71

PDA: potato dextrose agar. ^1^ With respect PDA-lactic acid (0.05 M). ^2^ Means ± standard deviation (*n* = 3).

**Table 3:** Chitosan sensitivity of other fungi species isolated from anthracnose on mango. Radial growth in medium PDA, PDA-lactic acid (0.05 M), and PDA-lactic acid (0.05 M) with 1 g L^−1^ chitosan at 25 °C.

Isolate/Specie	Radial Growth Rate: Log Phase (μm h^−1^)			Inhibition^1^ of Radial Growth (%) by Chitosan at 120 h
	PDA	PDA-Lactic Acid	PDA-Lactic Acid Chitosan	
004*/Alternaria* sp.	116 ± 2 ^a2^	60 ± 1 ^b^	35 ± 1 ^c^	20.28 ± 2.40 ^G^
009-1/*Alternaria* sp.	192 ± 3 ^b^	294 ± 3 ^a^	17 ± 4 ^c^	24.07 ± 2.62 ^G^
H6-1b/*Alternaria tenuissima*	76 ± 3 ^b^	155 ± 3 ^a^	24 ± 3 ^c^	72.72 ± 1.84 ^BC^
H2-2/*Fusarium* sp.	206 ± 1 ^b^	255 ± 2 ^a^	105 ± 7^c^	71.54 ± 0.00 ^BC^
H2-3/*Fusarium* sp.	277 ± 1 ^a^	228 ± 3 ^b^	23 ± 4 ^c^	87.62 ± 0.00 ^A^
H2-7/*Pestalotiopsis* sp.	578 ± 7 ^a^	497± 4 ^b^	159 ± 4 ^c^	67.18 ± 0.44 ^CD^
*H2-9/Pestalotiopsis* sp.	297 ± 1 ^a^	294 ±1 ^b^	87 ± 1 ^c^	74.87 ± 0.00 ^B^
H5-3/*Pestalotiopsis* sp.	40 ± 0 ^c^	158 ± 1 ^a^	59 ± 1 ^b^	43.33 ± 1.57 ^F^
H3-8/*Curvularia lunata*	223 ± 1 ^a^	186 ± 8 ^b^	55 ± 1 ^c^	65.22 ± 1.88 ^D^
008/*Diaporthe pseudomangiferae*	293 ± 6 ^a^	153± 7 ^b^	106 ± 8 ^c^	53.75 ± 0.0 ^E^
*H6-2/Epicoccum nigrum*	133 ± 2 ^a^	56 ± 3 ^b^	12 ± 1 ^c^	69.22 ± 1.71 ^BCD^

PDA: potato dextrose agar. ^1^ With respect to PDA-lactic acid (0.05 M). ^2^ Means ± standard deviation (*n* = 3). Values followed by different capital letters in columns and lowercase letters in rows are statistically different (Tukey *p* ≤ 0.05).

**Table 4:** Primer sequences for identification of fungal isolates.

Genetic Marker	Forward (5′-3′)	Reverse (5′-3′)	Reference
Internal transcribed spacers (ITS)	ITS1 (TCCGTAGGTGAACCTGCGG)	ITS4 (TCCTCCGCTTATTGATATGC)	[48,49]
β-tubulin 2 (β-Tub_2_)	Bt2a (GGTAACCAAATCGGTGCTGCTTTC)	Bt2b (ACCCTCAGTGTAGTGACCCTTGGC)	[48]
Glyceraldehyde-3-phosphate dehydrogenase (GAPDH)	GDF1 (GCCGTCAACGACCCCTTCATTGA)	GDR1 (GGGTGGAGTCGTACTTGAGCATGT)	[48]
Actin (Act)	ACT-512F (ATGTGCAAGGCCGGTTTCGC)	ACT-783R (TACGAGTCCTTCTGGCCCAT)	[48,49]
Calmodulin (CaM)	CL1C (GAATTCAAGGAGGCCTTCTC)	CL2C (CTTCTGCATCATGAGCT GAC)	[48]
Chitin synthase (CHS-1)	CHS1-79F (TGGGGCAAGGATGCTTGGAAGAAG)	CHS-1-354R (TGGAAGAACCATCTGTGAGAGTTG)	[48,49]
Apn2-Mat1-2 intergenic spacer (ApMat)	AMF1 (TCATTCTACGTATGTGCCCG)	AMR1 (CCAGAAATACACCGAACTTGC)	[50]

**Table 5:** Equations for determining the deacetylation degree and molecular weight of chitosan.

Number or Name of Equation	Equation
1 (DA: acetylation degree)	$\frac{A_{1320}}{A_{1420}}=0.3822+0.03133\;DA$
2 (DD: deacetylation degree)	DD=100−DA
Relative viscosity	η_rel_ = t_flux solution of chitosan/_t_flux of solvent_
Specific viscosity	η_sp_ = η_rel_ – 1
Huggins	[η] = η_sp_/C
Kramer	[η] = (ln η_rel_)/C
Mark–Houwink–Sakurada	[ƞ] = k (Mv)^α^

## Data Availability

They can be requested from the corresponding author.

## Sample Availability

Samples of the fungi isolates are available from the corresponding author.
